# Incidence and prevalence of gout in Western Sweden

**DOI:** 10.1186/s13075-016-1062-6

**Published:** 2016-07-13

**Authors:** Mats Dehlin, Panagiota Drivelegka, Valgerdur Sigurdardottir, Anna Svärd, Lennart T. H. Jacobsson

**Affiliations:** Department of Rheumatology and Inflammation Research, Institute of Medicine, Sahlgrenska Academy, University of Gothenburg, Gothenburg, Sweden; Rheumatology Clinic, Falun Hospital, Falun, Sweden

**Keywords:** Gout, Prevalence, Incidence, Urate lowering treatment

## Abstract

**Background:**

The aim of the present study was to describe prevalence and trends in the incidence of gout and patterns of urate-lowering treatment (ULT) in the Western Swedish Health Care Region (WSHCR) from 2002 to 2012.

**Methods:**

We used regional and national healthcare registers to estimate the prevalence and incidence of gout in 2012, and trends in incidence for each calendar year from 2005 to 2012. We also investigated the pattern of ULT for gout using the Swedish Prescribed Drug Register.

**Results:**

In 2012, in the population aged 20 years and above, the prevalence of gout was 1.8 % (95 % confidence interval (CI) 1.77 to 1.82) and the incidence was 190 cases (95 % CI 180 to 200) per 100,000 person-years. Applying more strict definitions for a gout case rendered a prevalence of 1.36 % (95 % CI 1.34 to 1.38) and 0.5 (95 % CI 0.49 to 0.51) per 100,000 person-years, respectively. The incidence of gout increased steadily and significantly from 2005 to 2012, with an almost 50 % increase in the total population. There was no significant difference in the prevalence of gout in rural compared to urban areas. ULT was dispensed to only 42 % of patients with gout in 2012 who had ever been diagnosed with gout during the preceding 10-year period.

**Conclusions:**

Gout is the most common arthritic disease in WSHCR, Sweden, and has increased substantially over the last decade, with only a minority of prevalent cases in 2012 receiving ULT.

**Electronic supplementary material:**

The online version of this article (doi:10.1186/s13075-016-1062-6) contains supplementary material, which is available to authorized users.

## Background

Gout is the most common form of inflammatory arthritis with a prevalence range of 1–4 % in North America and Western Europe [1–5]. Different approaches to identification of gout cases (diagnostic definitions) have been used in these studies, including International Classification of Diseases (ICD) codes in diagnosis registers and administrative claims databases [4, 6], ICD codes combined with hyperuricemia or gout treatment [1, 7], classification criteria [3], self-reported gout in home interviews [2], and gout diagnosis questionnaire performed by telephone interview [5, 8]. Recurrent episodes of pronounced acute inflammation due to deposition of monosodium urate crystals result in pain and frequently subsequent chronic arthritis with large disability for the individual and great costs for society. Gout is also closely associated with a number of comorbidities, including cardiovascular morbidity, and has been shown to be associated with an increased mortality [9, 10].

Several studies suggest an increasing incidence and prevalence of gout, for example in the UK and North America [4], which is not solely explained by longer survival of the general population. There are large geographical variations in prevalence which probably reflects cultural and genetic differences between populations [11]. This stresses the need for regional and national data. There is an almost complete lack of reports regarding prevalence and no studies on incidence of gout in Sweden or other Nordic countries [12–15]. Studies from the UK [1] and Italy [16] suggest variations in prevalence within countries, possibly reflecting differences in lifestyle. The present study addresses these issues within the western part of Sweden, an area which is representative of the country as a whole in terms of demographics, socioeconomic factors, and healthcare consumption [17, 18].

The aims of the present study were to assess in the Western Swedish Health Care Region (WSHCR): 1) the incidence and prevalence of gout in 2012; 2) possible differences in prevalence in rural and urban areas; 3) the proportion of patients having received a diagnosis of gout receiving urate-lowering treatment (ULT); and 4) trends in incidence of gout from 2005 through 2012.

## Methods

### Study design

This is a population-based study of incidence and prevalence rates using national healthcare registers.

### Setting and study population

All inhabitants aged 20 years and above in the WSHCR from 1 January 2002 to 31 December, approximately 20 % of the total population of Sweden, were included.

### Data sources

The Western Swedish Health Care Register (VEGA) was used to identify cases with gout and to determine the occurrence of diagnosed comorbidities. This register contains information about all healthcare contacts at inpatient and outpatient secondary clinics, as well as at primary care clinics. The register contains the date of contact and both primary and auxiliary diagnoses given by the treating physician according to the Swedish version of the ICD. Since 1997 the 10^th^ version of ICD (ICD-10) has been used in Sweden.

The Swedish Prescribed Drug Register [19] contains information about all prescribed drugs dispensed by Swedish pharmacies since July 2005. This register was used to determine dispensation of ULT as well as use of diuretics during 2012, using the appropriate Anatomical Therapeutic Chemical Classification System (ATC codes) (for more information see Additional file 1: Table S1).

Demographic data were obtained from Statistics Sweden [18], which holds data on immigration, emigration, and residency, as well as data on socio-economic factors (e.g., marital status and level of formal education) for all persons residing in Sweden.

### Case definition of gout

Gout was defined as having been given an ICD coded diagnosis of gout (M10 or M14) at a healthcare visit to a physician from 1 January 2002 through 31 December 2012. Gout was defined by three definitions: 1) liberal case definition requiring ≥1 visit in any care setting with a primary or auxiliary diagnosis of gout; 2) base case definition requiring ≥1 visit in any care setting with a primary ICD-10 diagnosis of gout; and 3) strict case definition requiring ≥2 visits with a primary ICD-10 diagnosis of gout in any care setting or ≥1 diagnosis of gout at a visit to a rheumatologist. The latter definition has been shown in our previous validation study to have a positive predictive value in relation to the Mexico and Netherlands classification criteria for gout of ≥80 % in Sweden [20].

### Definitions of comorbidities and ULT treatment

Common comorbidities were separately identified for prevalent cases by 31 December 2012 for those fulfilling the three case definitions for gout. Comorbidities were defined as the presence of at least one visit to a physician in primary or specialized care or a hospitalization with a corresponding ICD-coded diagnosis during 2002–2012 (for ICD-10 codes see Additional file 1: Table S1). The treatment with diuretics was defined as having at least one dispensed prescription during 2012 (for ATC codes see Additional file 1: Table S1).

ULT treatment during 2012 was defined in a similar fashion as having at least one dispensed prescription for any of the following drugs in 2012: allopurinol, febuxostat, and probenecid (for ATC codes see Additional file 1: Table S1).

Using the information from 2012 regarding those treated with ULT, we also calculated the prevalence that year for people fulfilling any of our three definitions for gout and having a dispensed prescription of ULT in 2012.

### Definitions of rural and urban municipality

Eighty-five percent of the Swedish population is living in urban conditions defined as a community with more than 200 inhabitants and a distance shorter than 200 meters between houses [21]. WSHCR consists of 49 municipalities with populations ranging from 4665 to 526,089. In nine of the 49 municipalities in WSHCR, ≥85 % of the population live in an urban environment and, in the remaining 40 municipalities, ≤81 % of the population live in an urban environment [21] (for more information see Additional file 2: Figure S1).

### Statistical analysis

Prevalence was calculated using the number of people aged 20 years and above fulfilling our case definitions for gout (liberal, base, strict), overall and with ULT treatment, who were alive and living in WSHCR by 31 December 2012 as the numerator and the total population aged 20 years and above in WSHCR by 31 December 2012 as the denominator.

Incidence was calculated using the number of incident gout cases aged 20 years and above per calendar year as the numerator and the total person-years occurring in the population aged 20 years and above in WSHCR at the same year as the denominator. The incidence of gout in 2012 was defined as the number of patients that received a primary or auxiliary diagnosis of gout in 2012, the liberal case definition, with no recorded diagnosis of gout in the preceding 10 years (2002–2011). When defining incidence for analyses of trends we arbitrarily defined an incident case as a patient not having had a visit with a recorded primary or auxiliary (liberal case definition) diagnosis of gout during the preceding 3 calendar years in order to ensure comparability between the different years; thus, this was analysis of trends performed between 2005 and 2012. A sensitivity analysis requiring a 5-year period free of gout diagnosis, analyzing trends in incidence for the period 2007–2012, was also performed.

Descriptive statistics were used to summarize the demographic characteristics. We calculated both crude and standardized estimates (direct method) using the whole Swedish population aged ≥20 years in 2012 as the standard population when comparing the prevalence in rural and urban municipalities and when comparing incidence rates by year in the analyses of trends. The significance of linear incidence trend was performed using logistic regression models. All statistical analyses were performed using SAS 9.4.

## Results

### Prevalence and ULT treatment

**Fig. 1:**
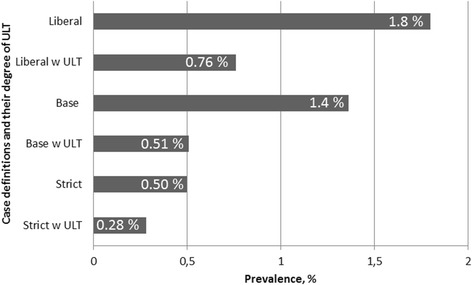
Prevalence of gout in WSHCR at 31 December 2012 in people aged ≥20 years by different case definitions and degree of urate-lowering treatment (*ULT*). *w* With

We identified 30,430 individuals aged 20 years and above with ≥1 ICD-10 diagnosis (primary or auxiliary) of gout in WSHCR from 1 January 2002 to 31 December 2012. Out of these, 8007 individuals had either emigrated or died by the end of 2012, leaving a total of 22,423 living individuals in the area in 2012 (for more information and flowchart see Additional file 2: Figure S1). This corresponds to crude point prevalence in 2012 according to the liberal, base, and strict definitions for gout of 1.8 %, 1.4 %, and 0.5 %, respectively (Fig. 1).

ULT was only dispensed to a minority of the gout patients in 2012, with a period prevalence of 42 %, 37 %, and 55 % for patients fulfilling the liberal, base, and strict case definitions (Fig. 1). The vast majority was prescribed allopurinol, less than 2 % received probenecid, and febuxostat was not used at all in 2012.

The characteristics of gout cases, whether using the liberal, base, or strict definition, were similar overall (Table 1). There was a male predominance (range 70–79 %) and the mean age was 68–69 years. There were high frequencies of comorbidities known to be associated with gout (Table 1) that were similar irrespective of gout case definition, with approximately 63–68 % of the cases having a previous diagnosis of hypertension, 25–27 % of ischemic heart disease, 13–14 % of stroke, 20–23 % of diabetes mellitus, 17–20 % of renal disease, and 20–21 % having concurrent medication with thiazide diuretics (Table 1).

**Table 1:** Prevalence of gout according to the liberal, base, and strict case definitions with patient characteristics for the three groups

	Liberal case definition *n* = 22,243	Base case definition *n* = 16,833	Strict case definition *n* = 6184
Total population	1,237,935		
Total prevalence (95 % CI)	1.8 (1.77–1.82)	1.36 (1.34–1.38)	0.5 (0.49–0.51)
Sex (male), %	70 %	72 %	79 %
Age 2012 (years), mean (SD)	69 (14)	68 (14)	68 (14)
Level of education^a^			
9 years or less	47 %	45 %	45 %
9 to 12 years	38 %	39 %	40 %
More than 12 years	15 %	16 %	15 %
Co-morbidities			
Hypertension	68 %	63 %	67 %
Diabetes	23 %	20 %	21 %
Ischemic heart disease	27 %	25 %	28 %
Congestive heart failure	20 %	18 %	22 %
Stroke	14 %	13 %	14 %
Renal disease	19 %	17 %	20 %
Diuretic treatment			
Thiazide	21 %	20 %	20 %
Loop	41 %	37 %	37 %
Potassium conserving diuretics	22 %	20 %	20 %

^a^4 % missing data on level of education in the liberal case definitions and 2 % missing in base and strict case definition

*CI* confidence interval, *SD* standard deviation

**Fig. 2:**
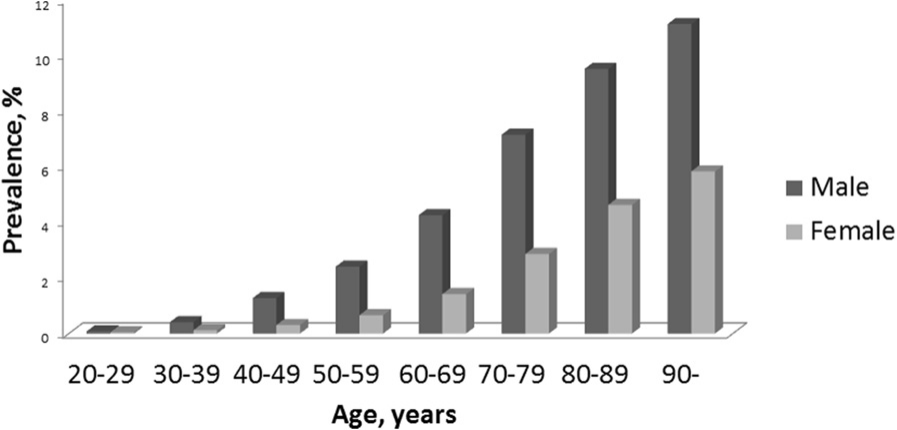
Prevalence of gout in WSHCR at 31 December 2012 in people age ≥20 years by age and sex according to the liberal case definition

The prevalence of gout in WSHCR was higher in men and increased with age, with a prevalence of more than 3 % in men aged 50–59 years and almost 7 % in men aged 70–79 years (Fig. 2). The sex difference in prevalence was most pronounced in those aged 30 to 59 years, with a male to female ratio of 3:1 to 4:1, which decreased to a two-fold increase in patients above 70 years (Fig. 2).

### Prevalence in urban and rural areas

The age- and gender-standardized [18] prevalence of gout was slightly higher in the municipalities with more rural milieu (1.73 %) compared to the ones with a more urban environment (1.67 %), although this difference was not significant (for map illustrating the geographical location see Additional file 3: Figure S2).

### Incidence

The incidence of gout in 2012, demanding 10 preceding years (2002–2011) with no recorded diagnosis of gout, was 190 cases per 100,000 person-years (Table 2). The male to female ratio was more than 3:1 in those aged below 70 years, whereas it was only 2:1 to 3:1 in those aged above 70 years (Table 2).

**Table 2:** Incidence of gout in the Western Swedish Health Care Region in 2012 age ≥20 years per 100,000 person-years with 95 % confidence intervals overall, and by sex and by age groups

Group	Male		Female	
	*n*	Incidence rate (95 % CI)	*n*	Incidence rate (95 % CI)
Overall	1639	267 (260–280)	747	116 (110–130)
20–29 years	20	18 (10–30)	9	8 (0–20)
30–39 years	74	71 (60–90)	16	16 (10–30)
40–49 years	177	158 (140–180)	51	47 (40–60)
50–59 years	273	277 (250–310)	76	78 (60–100)
60–69 years	438	457 (420–500)	145	151 (130–180)
70–79 years	370	654 (590–720)	190	302 (260–350)
80–89 years	235	865 (760–980)	196	346 (300–400)
90 years and above	52	1168 (890–1530)	64	598 (470–760)

*CI* confidence interval

**Fig. 3:**
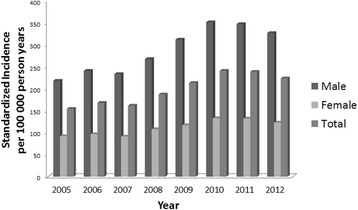
Incidence of gout in WSHCR 2005–2012 in people aged ≥20 years in total and by sex per 100,000 person-years, standardized for age and sex using the Swedish population as standard

The incidence of gout increased steadily and significantly from 2005 to 2012, with an almost 50 % increase in the total population (*p* = 0.0014; Fig. 3). The male to female ratio of gout incidence was consistently higher than 2:1 for all the years 2005 to 2012 (Fig. 3). In a sensitivity analysis, we also calculated incidence trend with a requirement of 5 preceding years without gout diagnosis, which resulted in similar results with a significant increase in incidence from 2007 to 2012 (*p* = 0.019).

## Discussion

In the present study we found an overall prevalence of ever-diagnosed gout in WSHCR Sweden in 2012 of 1.8 %, and 0.5 % when using a more stringent definition for disease. The incidence in 2012 was 190 per 100,000 person-years, with a significant increase over the previous decade. There was no significant difference in prevalence between urban and rural areas. In 2012, only a minority of the patients were treated with ULT.

The occurrence of gout has only scarcely been reported from Sweden and the neighboring Nordic countries [12–15, 22]. Studies from the rest of Europe have reported a variation in prevalence, with 2.5 % being reported in the UK for 2012 [1], 1.4 % in Germany for 2000 through 2005 [23], 0.9 % in France for 2013 [5, 8], and 0.9 % in Italy for 2009 [16]. Differences between studies may reflect different case ascertainment or true variation, possibly related to cultural and genetic differences between populations. In the present study we also applied a more strict method for defining gout, requiring two visits with a gout diagnosis in any care setting or one visit with such a diagnosis to a rheumatologist, by which we only identified approximately 25 % of the cases identified with the liberal definition. The strict definition has previously been validated by us and has been shown to have a positive predictive value for fulfilling classification criteria for gout of over 80 %, which was considerably higher compared to the positive predictive value for gout according to our liberal definition [20]. This reflects more frequent visits to the healthcare system with a diagnosis of gout by those defined by the strict definition and is likely to mirror a more severe disease phenotype. Although this supports the validity of our strict definition, it should be emphasized that classification criteria are intended to be used for classification in research studies and not for individual clinical diagnosis. This holds true not least for the 1977 ARA criteria for gout [24, 25]. The group defined according to the strict definition exhibited similar patterns of comorbidities and educational level as those defined according to the liberal or base definitions, but received ULT to a greater extent. This is compatible with the view that our liberal definition indeed represents true gout, but also that those defined according to the strict definition represent a more severe phenotype. Differences in ULT between the liberal and strict group may reflect that insufficient gout treatment is more pronounced in those with less severe disease. The three case definitions represent three levels of diagnostic certainty. A higher level of diagnostic certainty (as used in our strict definition) is mandatory when causal relationships or etiology to disease should be investigated. Although having high specificity, such a definition may have low sensitivity for detecting the whole spectrum of disease, a problem we addressed by also using a liberal definition for gout. Since several recent studies on rheumatoid arthritis from Sweden have only reported prevalence figures of 0.7 %, our findings indicate that gout is the most common form of arthritis in Sweden [26, 27].

Few studies have addressed the incidence of gout in the world, and there are no contemporary studies from Sweden and the Nordic countries. A Finnish study from 1974 reported 12 cases per 100,000 person-years with no incident cases in women [22]. In our study, the incidence of gout in women was 116 per 100,000 person-years. Surprisingly, we identified 76 women aged 20–49 years, thus possibly premenopausal, with incident gout, which is similar to results in the UK in 2012 [1]. Considering the conception that gout is very rare in people of this age, these cases would have benefited from specific validation, which was not possible as part of this register study. The gout incidence in 2012 of 190 per 100,000 person-years in our study was also similar to that reported in the UK [1], but was about twice as high as that reported from Italy [23]. We found an almost 50 % increase in the incidence of gout from 2005 to 2012, results which are similar to those reported in the UK [1]. This probably reflects a true increase of the disease in Sweden, although it may also be partly influenced by changes in diagnostic recording routines over time.

Geographical variation in the prevalence of gout has been reported from the UK [1] and Italy [16]. In the UK study, the differences were suggested to be related to differences in socioeconomic status, lifestyle, and nutrition. A north–south prevalence gradient was observed in the Italian study, and was suggested to be attributed to different dietary habits related to the Mediterranean diet. In our study, we found a no difference in prevalence in rural compared to more urban municipalities. Older studies from the UK and Finland have, on the other hand, reported a higher risk in urban areas compared to rural ones [14, 28].

Thirty-seven to 55 % of the gout patients in the study were on ULT treatment in 2012, using the base and strict case definition, respectively. These figures are low, especially considering the growing number of recommendations to treat gout with ULT earlier in the disease course. Studies from the UK [1] and Taiwan [29] confirm that this is a widespread problem, although it is unclear how large the proportion of patients with gout is that ideally should be treated. Further studies are needed to explore to what extent these low figures are due to poor compliance by patients or to shortcomings of the healthcare system.

There are several possible limitations of our study. First, our case definitions were based on diagnoses of gout made in the clinical situation rather than according to the different proposed classification criteria [30–35], which may have led to misclassification bias. We have, however, previously validated our strict case definition [20] and found it to have a high predictive value for fulfilling classification criteria for gout. In addition to this definition, we chose to report on ever-diagnosed gout, the liberal case definition, to increase comparability to previous studies. Second, gout has an intermittent course with possibly long clinically silent phases which may hamper any attempt to find the true occurrence of the disease, a problem that may be of less importance in the present study since we had a long observation period. Furthermore, sensitivity analyses using different assumptions in the analyses of incidence trends resulted in similar estimates supporting the robustness of our results. Third, the study was performed in the western part of Sweden which may not be nationally representative. However, other studies have demonstrated very similar statistics with regard to sociodemographic distribution and healthcare seeking in this region compared to Sweden as a whole [17, 18].

There are also several strengths of the present study. First, using the healthcare registers in the Swedish setting with virtually complete coverage makes the results population representative. Second, loss to follow-up is a minor problem since death or emigration are reliably followed in this population by the central statistics in Sweden. Third, the estimates for ULT were retrieved from an independent data source.

## Conclusion

In conclusion, we found an increasing incidence of gout over the last decade with a prevalence in WSHCR of 1.8 % in 2012. According to a more stringent definition, probably reflecting a more severe phenotype, only 0.5 % had gout. Irrespective of definition, management of gout measured by the degree of ULT in 2012 was as poor as that reported in other European countries previously.

## Abbreviations

ATC, Anatomical Therapeutic Chemical Classification System; CI, confidence interval; ICD, International Classification of Diseases; ULT, urate-lowering treatment; VEGA, Western Swedish Health Care Register; WSHCR, Western Swedish Health Care Region

## Additional files

Additional file 1: Table S1. Definition of co-morbidities by ICD-10 codes and ULT and diuretics by ATC codes. (DOCX 93 kb) Additional file 2: Figure S1. Flowchart of gout patients who were identified, migrated, and died. (TIF 379 kb) Additional file 3: Figure S2. Map of WSHCR with municipalities with ≥85 % of their population living in urban environment (*dark grey*) and the municipalities with ≤81 % of their population living in urban environment (*light grey*). (TIF 284 kb)
