# Supplementary material for: Incidence and prevalence of gout in Western Sweden
Source: Arthritis Res Ther. 2016 Jul 13;18:164. doi: 10.1186/s13075-016-1062-6 (PMC4944470; doi:10.1186/s13075-016-1062-6)
Supplement: Additional file 1: Table S1. — Definition of co-morbidities by ICD-10 codes and ULT and diuretics by ATC codes. (DOCX 93 kb) [file 13075_2016_1062_MOESM1_ESM.docx]

| Co-morbidity | ICD-10 code | ATC-code |
| --- | --- | --- |
| Hypertension | I10-15 |  |
| Diabetes | E10-14, O24 |  |
| Ischemic Heart Disease | I20-25 |  |
| Congestive heart failure | I 50 |  |
| Stroke | I60-64, G45 |  |
| Renal disease | N00-08  N11-22 |  |
| ULT and diuretics | | |
| Allopurinol |  | M04AA01 |
| Probenecid |  | M04AB01 |
| Febuxostat |  | M04AA03 |
| Diuretics, tiazid |  | C03A-B  C07BB  C09BA02-03  C09BA05  C09BA06  C09BA08  C09BA09  C09BA15  C09CA01  C09DA01  C09DA02-4  C09DA06-7  C09XA52  C09XA54 |
| Diuretics, loop |  | C03C |
| Potassium conserving diuretics |  | C03D-E |

Additional file 1: Table S1 – Definition of co-morbidities by ICD10-codes and ULT and diuretics by ATC-codes.
