# Supplementary figures and images for: Incidence and prevalence of gout in Western Sweden
Source: Arthritis Res Ther. 2016 Jul 13;18:164. doi: 10.1186/s13075-016-1062-6 (PMC4944470; doi:10.1186/s13075-016-1062-6)

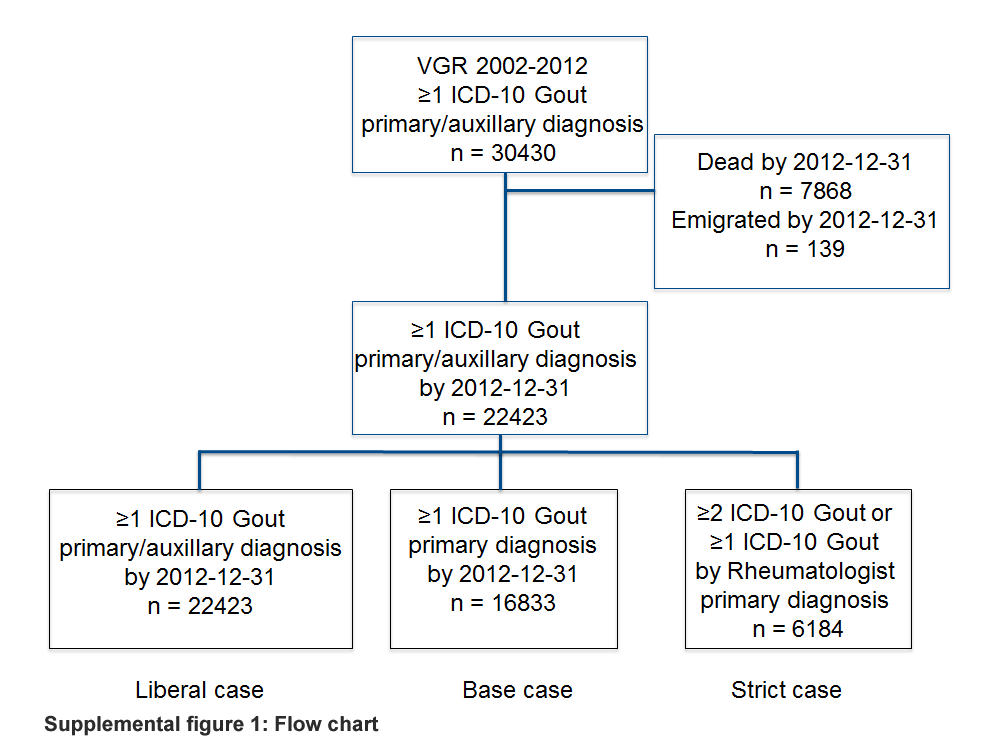

Supplement: Additional file 2: Figure S1. — Flowchart of gout patients who were identified, migrated, and died. (TIF 379 kb) [file 13075_2016_1062_MOESM2_ESM.tif]
